# Resting energy expenditure, body composition, and metabolic alterations in breast cancer survivors vs. healthy controls: a cross-sectional study

**DOI:** 10.1186/s12905-024-02900-y

**Published:** 2024-02-12

**Authors:** Timia Van Soom, Wiebren Tjalma, Ulrike Van Daele, Nick Gebruers, Eric van Breda

**Affiliations:** 1 Faculty of Medicine and Health Sciences, Department of Rehabilitation Sciences & Physiotherapy, Research group MOVANT, Multi-disciplinary Metabolic Research Unit (M2RUN), University of Antwerp, Universiteitsplein 1, 2610 Antwerp, Belgium; 2 Antwerp University Hospital (UZA), Multidisciplinary Breast Clinic, Wilrijkstraat 10, 2650, Edegem, Belgium; 3 Antwerp University Hospital (UZA), Multidisciplinary Edema Clinic, Wilrijkstraat 10, 2650 Edegem, Belgium; 4 Department of Medicine of University of Antwerp, Faculty of Medicine and Health Sciences, Universiteitsplein 1, 2610 Antwerp, Belgium; 5 OSCARE, Organization for Burns, Scar Aftercare and Research, Van Roiestraat 18, 2170 Antwerp, Belgium

**Keywords:** Breast cancer, Chemotherapy, Resting energy expenditure, Body composition, Indirect calorimetry, Metabolic dysfunction

## Abstract

**Purpose:**

This study aimed to investigate the difference in absolute and fat free mass (FFM)-adjusted resting energy expenditure (mREE) and body composition (body weight, fat mass (FM), FFM) between breast cancer survivors (BCs) and controls. Correlations with body composition were analyzed. We examined if survival year, or being metabolically dysfunctional were predictive variables.

**Methods:**

A cross-sectional analysis was conducted on 32 BCs ≤5 years post treatment and 36 healthy controls. Indirect calorimetry measured absolute mREE. Body composition was determined by BOD POD. FFM-adjusted mREE was calculated (mREE/FFM). The Harris-Benedict equation was used to predict REE and determine hyper−/hypometabolism (mREE/pREE). The database of the multidisciplinary breast clinic of the University Hospital of Antwerp was consulted for survival year and metabolic dysfunctions.

**Results:**

BCs have similar absolute mREE and greater FFM-adjusted mREE compared to controls. Absolute mREE and body composition between BCs differed; adjusted mREE was similar. FFM correlated significantly with absolute mREE in BCs. A significant interaction term was found between survival year and FM for absolute mREE.

**Conclusion:**

BCs have similar absolute mREE, but higher FFM-adjusted mREE. Differences in body composition between BCs are suggested to cause inter-individual variations. We suggest that increased FFM-adjusted mREE is caused by metabolic stress related to cancer/treatment. Accurate measurement of REE and body composition is advised when adapting nutritional strategies, especially in patients at risk for developing metabolic dysfunctions.

**Supplementary Information:**

The online version contains supplementary material available at 10.1186/s12905-024-02900-y.

## Introduction

Breast cancer is the most prevalent tumor type in women, representing 30% of the annual cancer incidence [1]. Advancements in screening procedures and (neo) adjuvant therapies are credited with improved survival rates [2]. Chemotherapeutic agents are administered in 56% of the patients with breast cancer stage (II)III-IV as standalone treatment, or in combination with other therapies [3]. Despite the therapeutic effects, a variety of adverse side-effects have been recorded after treatment. Amongst changes in body weight (BW) and body composition (fat mass (FM); fat free mass (FFM)), deteriorating metabolic profiles are often present in women with breast cancer, from time of diagnosis throughout the rest of the patient’s life (i.e. breast cancer survivors (BCs)), which elevates the risk for developing the metabolic syndrome [2].

Metabolic dysfunction is the hallmark of the metabolic syndrome with clinical features such as excess accumulation of adipose tissue and obesity, hypertension, dyslipidemia, insulin resistance, and increase in blood glucose [4]. Metabolic disturbances are common in BCs after treatment, with merging evidence directing towards chemotherapy (CT) as potential cause [5]. Often during CT, changes in FM and FFM occur, which are associated with toxicity-induced comorbidities [6]. In terms of FM, the influence of CT on accumulating visceral and ectopic adipose tissue is rapid (within 2–3 months) and persistent (> 1 year) [4]. Chemotherapeutic agents also induce oxidative stress, increasing the risk for developing metabolic dysfunctions [2]. The presence of ≥2 metabolic dysfunctions is related to cancer development and –recurrence [7]. Metabolic alterations and changes in FM and FFM often go hand in hand, ultimately affecting the body’s energy needs [8, 9].

Indirect calorimetry (IC) is widely acknowledged as gold standard for measuring (resting) energy expenditure (m(R)EE; absolute mREE) [9, 10]. In health, BW seems to be the most decisive factor for REE due to the amount of FFM, consisting of organ tissue, bone tissue, and muscle tissue, and is highly metabolically demanding [11]. In disease, deflections in FFM-adjusted mREE (mREE/FFM) seem to represent metabolic stress, with increasing levels proportional to disease severity [8].

In cancer, both an increase (hypermetabolism) and decrease (hypometabolism) in the level of REE can occur, as well as no change at all [12]. The high variability is most likely due to the extensive range of clinical phenomena (cancer type, −stage, treatment, metabolic derangements) that can alter a patients’ metabolic demand. In this regard, repeated measurements of REE in combination with body composition analysis over the course of different illness trajectories have provided more insight in metabolic alterations, as dynamic patterns were unraveled suggested to correspond with illness progression or resolution [13–16]. The use of IC to measure REE is therefore preferred over pREE to accurately assess the metabolic demand and determine nutritional goals in patient populations [12].

The clinical value of absolute and adjusted mREE in relation to body composition and the presence of metabolic stress related to metabolic dysfunctions in different patient populations has already been acknowledged [14, 16, 17]. In BCs post treatment however, evidence is conflicting as a large heterogeneity exists within the population. Therefore, the purpose of this study was 1) to explore the difference in absolute mREE, FFM-adjusted mREE and body composition parameters: FM, FFM, and BW in BCs compared to healthy controls; 2) to examine the correlations between body composition parameters and absolute mREE in BCs and controls to predict the individual contribution of body composition to absolute mREE; and 3) to investigate if survival time, and being metabolically dysfunctional (≥2 metabolic dysfunctions) are predictive variables for absolute mREE in BCs.

## Methods

This cross-sectional study was conducted at the Multidisciplinary Metabolic Research Unit (M^2^RUN) of Movant Research group; University of Antwerp (*Belgium*). Approval was given by the medical ethical committee of the University of Antwerp/Antwerp University Hospital (B300201837317).

### Participants

The database of the Multidisciplinary Breast Clinic of the University Hospital of Antwerp was consulted to find BCs meeting the eligibility criteria. Simultaneously, a healthy control group was recruited after advertising on social platforms (Facebook, Instagram, Twitter) or by direct contact. Eligible subjects were contacted by telephone and provided with an appointment at the laboratory. All participants signed informed consent at the day of the appointment. All study visits took place between November 25th, 2021 and September 17th, 2022.

### Breast cancer survivors

Female BCs between 40-65y were contacted. To represent the whole survivor group, women were included when they were either 1-, 3-, or 5 years post active treatment (survival time). To limit bias, age, BH, and BW were matched between survival years. Patients were controlled for tumor type (infiltrating ductal breast carcinoma, stage III – IV) and active treatment regimen (12 × 1 time/week taxol therapy; 4 × 1/2 weeks AC therapy; surgery; radiotherapy). Only women with a first diagnosis of BC were included. Subjects were excluded when one of the inclusion criteria was not met (Table 1).

**Table 1:** Demographic and anthropometric variables of participants and matching procedures

Group(n)/ Parameter	BCs (*n* = 32) Mean ± SD	Controls (*n* = 36) Mean ± SD	*p*-value
**Age (y)**	55.6 ± 7.4	52.9 ± 5.4	0.083
**Body Height (cm)**	167.9 ± 4.4	164.4 ± 6.4	0.750
**Body weight (kg)**	70.9 ± 14.9	70.4 ± 13.8	0.889
**Body composition**			
FM (kg)	26.4 ± 10.7	26.0 ± 13.7	0.879
FM (%)	35.3 ± 8.5	33.3 ± 8.5	0.344
FMi (FM/m^2^)	15.7 ± 6.3	15.4 ± 7.9	0.885
FFM (kg)	44.7 ± 6.0	46.1 ± 5.4	0.164
FFM (%)	63.1 ± 10.5	65.1 ± 11.8	0.471
FFMi (FFM/m^2^)	15.8 ± 1.7	16.4 ± 1.5	0.140
**BMI**	25.1 ± 4.9	25.1 ± 4.3	0.975
< 18.5: under – n (%)	1 (3.1%)	0 (0%)	
18.5–24.9: normal – n (%)	15 (46.9%)	19 (52.8%)	
25.0–29.9: over – n (%)	12 (37.5%)	12 (38.9%)	
> 30.0: obese – n (%)	4 (12.5%)	3 (8.3%)	
**Therapy n (%)**			N/A
No therapy	7 (21.9%)	36 (100%)	
Hormone therapy	25 (78.1%)	N/A	
**pREE**	1379 ± 158	1385 ± 134	0.865
**mREE**	1452 ± 171	1422 ± 153	0.452
**mREE/FFM**	33 ± 3	31 ± 3	0.031*
**mREE/FFMi**	92 ± 8	87 ± 10	0.028*
**Metabolism (%)**			N/A
Hyper	13 (40.6%)	6 (16.7%)	
Hypo	2 (6.3%)	2 (5.6%)	
Normo	17 (53.1%)	28 (77.8%)	
**Metabolically**			N/A
**(dys)functional n (%)**			
Metabolically functional	13 (40.6%)	36 (100%)	
Metabolically dysfunctional	19 (59.4%)	N/A	

*BCs* Breast cancer survivors, *SD* Standard deviation, *n* Number of participants, *y* Year, *cm* Centimeter, *kg* Kilogram, *mREE* Absolure resting energy expenditure*, pREE* Predicted resting energy expenditure, *mREE/FFM* fat free mass-adjusted mREE *mREE/FFMi* fat free mass index-adjusted mREE, *BMI* Body mass index, *FMi (FM/m*^*2*^*)* Fat mass index, *FFMi (FFM/m*^*2*^*)* Fat free mass index. *denotes significance at α < 0.05

### Metabolic (dys) function in BCs

BCs were additionally assigned to a metabolically functional (Funct) and dysfunctional (Dysf) group based on information extracted from the medical file. Relevant information was related to the presence of metabolic dysfunctions. Survivors were considered as dysfunctional when ≥2 of the following clinical abnormalities were present for > 6 months: 1) Dyslipidemia (hypercholesterolemia and hypertriglyceridemia) or medication, 2) hypertension or medication, 3) insulin resistance or medication, 4) increased adiposity (FM > 30% of total BW, with BMI > 25.0 kg/m^2^), or 5) increased blood glucose. The specific criteria for determining metabolic dysfunctions are added supplementary (Table A).

### Healthy controls

Women enrolled when they were between 40-65y old and in general good health: Absence of metabolic diseases that could alter REE (hyper−/hypothyroidism, burn wounds, liver diseases), < 2 metabolic dysfunctions (hypertension, hypertriglyceridemia, hypercholesterolemia, insulin resistance, increase in blood glucose), no common cold or flu < 2 weeks ago, no surgery < 1 month ago, no current use of pharmaceuticals (medication of any type), and no (history of) cancer (any type). Participants were excluded if one of the criteria was unmet. Healthy subjects were matched with BCs for age, BH, and BW (Table 1).

## Procedures and outcome measures

### Setting

Participants were invited for a single appointment (1.5 hr), on which all procedures for data collection were done consecutively. Assessments were executed in the morning (7h00am-11h30am). Subjects were not allowed to eat < 8 hrs before measurement, while drinking water was allowed freely until 2 hr. prior to the appointment. Participants were asked to refrain from heavy exercise 24 hr. before measurements.

### Demographic and anthropometric variables

The following demographic and anthropometric data were collected: pREE (*HBeq* = 447.593 + (9.247 *x BW*(*kg*)) + (3.098 *x BH*(*cm*)) − (4.330 *x age*(*y*))), survival year, current cancer-related endocrine treatment (Table B. appendix), age(y), BW (kg), FM (kg,%) FFM (kg,%) BH (cm), and body mass index (BMI; $\frac{BW(kg)}{BL(m)2}$) to evaluate the obesity status (underweight (BMI < 18.5), normal weight (BMI = 18.5–24.9), overweight (BMI = 25.0–29.9), obese (BMI > 30.0)). To assess body composition in relation to body size, fat mass index (FMI; $\frac{FM(kg)}{BL(m)2}$) and fat free mass index (FFMI; $\frac{FFM(kg)}{BL(m)2}$) were calculated.

### Indirect calorimetry

IC measurements were done with an open circuit diluted flow calorimeter (*Omnical IV*, *Maastricht Instruments*, The Netherlands). Calibration of the device was performed automatically every 30 min with span gas (18% O_2_ and 0.8% CO_2_) and nitrogen gas (100%). Validation of the system by methanol combustion was performed weekly [18]. All measurements were normalized to standard temperature and pressure dry (STPD) values by measuring temperature, humidity, and pressure.

The measurements were executed in a basic version of a respiratory room (14m^3^). Participants were instructed to lay in a semi-inclined position while staying awake. Minimal activity (reading, desk work, listening to music) was allowed. The room served as reservoir collecting $\dot{\textrm{V}}$ O_2_ and $\dot{\textrm{V}}$ CO_2_. The measurement lasted 60 min and data were provided every minute. Results of the last 50 min were used. $\dot{\textrm{V}}$ O_2_ and $\dot{\textrm{V}}$ CO_2_ were converted to REE values (Weir, 1949). Data were collected as kcal/min and recalculated to kcal/24 hrs (absolute mREE) to determine FFM-adjusted mREE adjusted ($\frac{mREE}{FFM}$). The metabolic status was defined by the % difference between pREE and mREE (hypermetabolism (mREE/pREE> + 10%), hypometabolism (mREE/pREE<− 10%), normometabolism (mREE/pREE = ±10%)).

### Bod pod

FM and FFM were assessed by air-displacement plethysmography (BOD POD, *Cosmed*, United States) [19]. The device was calibrated prior to each analysis, as prescribed by the manufacturer. A warm-up period of 30 min was followed by a 15 min quality control of hardware, scale, and accuracy and reliability of volume assessment. For the assessment (10 min), participants were asked to remove all accessories and wear snug fitting clothing and swim cap to limit the effects on body surface area. A precise measurement of BW was followed by 2–3 (depending on individual fidgeting) analyses of body volume to determine body density ($Body\ density=\left(\frac{body\ weight}{body\ volume}\right)$). The relative contribution of FM to body weight (FM%) was calculated by the SIRI equation: $FM\%=\left(\frac{495}{body\ density}\right)-450$. Next, the relative contribution of FFM to body weight (FFM%), total amount of FM (kg) and FFM (kg) were derived.

### Statistical analysis

Quantitative variables are expressed as mean ± SD. All variables were checked for normality (Kolmogorov-Smirnov, QQ-plot and histogram) and were parametric. Characteristics of the study participants include: age, BH, BW, pREE, obesity status, current hormone therapy, metabolic status, and being metabolically (dys)functional; metabolic parameters include: absolute mREE and FFM-adjusted mREE; parameters related to body composition include: FM, FFM, FMi, FFMi, and BW. The difference in mean of all continuous variables between controls and BCs was tested by independent samples T-test. To investigate if differences were present between Dysf BCs, Funct BCs, and healthy controls, analysis of variance (ANOVA) was conducted with post-hoc Dunnett correction. The correlation between body composition parameters, and metabolic parameters in overall BCs, and healthy controls is expressed by Pearson correlation coefficient (r). Multiple linear hierarchical regression models were fitted to estimate the contribution of the significantly correlated body composition parameters to absolute mREE in overall BCs, and controls. Body composition parameters were entered stepwise in the regression model with the highest significantly correlated parameter first. Variables that intercorrelated were not considered in the model due to multicollinearity. The proportion of variance of the dependent variable that is explained by the independent variable is expressed by the R^2^ change. In addition, univariate analysis was conducted to fit similar multiple regression on absolute mREE in BCs when accounting for FM and/or FFM for following categorical variables: Survival year, being metabolically (dys)functional. Significant interaction terms with body composition parameters are shown. The contribution of the individual variables is expressed by the partial Eta^2^ (η_p_^2^). All statistical tests are executed two-sided (Significance: α < 0.05). Statistical analyses were performed with SPSS software (SPSS v29, IBM Business Analytics, New York, USA).

## Results

In total, 287 BCs were screened for eligibility (72 women in 1-year survival; 83 in 3-year survival; 132 in 5-year survival). 53 (1y), 47 (3y), and 87 (5y) BCs were contacted for study participation.

The main reasons for refusal were time constraints. Demographic and anthropometric data of all women (*n* = 68) enrolled are presented in Table 1. The control group consisted of 36 women, while the BCs group included 32 women (10 (1y); 11 (3y); 11 (5y) survival). Matching procedures between BCs and controls are found in Table 1.

### Resting energy expenditure and body composition in BCs and healthy controls

No significant differences were found in absolute mREE (BCs: 1452 ± 171 vs. controls: 1422 ± 153; *p* = 0.452). Absolute mREE between Funct BCs, Dysf BCs, and healthy controls, was significantly different (*p* = 0.049). Dysf BCs had significantly higher levels of absolute mREE compared to Funct BCs (Dysf: 1506 ± 174 vs. Funct: 1372 ± 116; *p* = 0.026) (Table 2).

**Table 2:** ANOVA: Comparison of means between dysfunctional BC survivors, functional BC survivors and healthy controls

Group(n)/ Parameter	Dysf BCs (*n* = 19) Mean ± SD	Funct BCs (*n* = 13) Mean ± SD	Controls (n = 36) Mean ± SD	p-value (Dysf. vs. control) (Funct. vs. control) (Dysf. vs. funct)
**mREE**	1506 ± 174	1372 ± 116	1422 ± 153	0.049*
				0.115 (Dysf. vs. control)
				0.531 (Funct. vs. control)
				0.026* (Dysf. vs. funct)
**mREE/FFM**	32 ± 3	33 ± 3	31 ± 3	0.080
				0.213 (Dysf. vs. control)
				0.084 (Funct. vs. control) 0.576 (Dysf. vs. funct)
**mREE/FFMi**	92 ± 8	92 ± 8	87 ± 10	0.098
				0.120 (Dysf. vs. control)
				0.129 (Funct. vs. control)
				0.995 (Dysf. vs. funct)
**Body weight (kg)**	77.2 ± 14.9	61.6 ± 9.2	70.4 ± 13.8	0.008*
				0.145 (Dysf. vs. control)
				0.090 (Funct. vs. control)
				0.002* (Dysf. vs. funct)
**Body composition**				
FM (kg)	31.4 ± 10.7	20.1 ± 6.8	26.0 ± 13.7	0.040*
				0.259 (Dysf. vs. control)
				0. 209 (Funct. vs. control)
				0.002* (Dysf. vs. funct)
FM (%)	37.9 ± 8.8	31.7 ± 6.6	33.3 ± 8.5	0.081
				0.115 (Dysf. vs. control)
				0.769 (Funct. vs. control)
				0.044* (Dysf. vs. funct)
FMi (FM/m^2^)	18.3 ± 6.2	11.9 ± 4.1	15.4 ± 7.9	0.041*
				0.265 (Dysf. vs. control)
				0.210 (Funct. vs. control)
				0.003* (Dysf. vs. funct)
FFM (kg)	46.8 ± 6.1	41.8 ± 4.2	46.1 ± 5.4	0.029*
				0.884 (Dysf. vs. control)
				0.033* (Funct. vs. control)
FFM (%)	61.7 ± 8.1	65.1 ± 12.9	65.1 ± 11.8	0.013* (Dysf. vs. funct)
				0.548
				0.495 (Dysf. vs. control)
				1.000 (Funct. vs. control)
FFMi (FFM/m^2^)	16.4 ± 1.9	15.0 ± 1.0	16.4 ± 1.5	0.382 (Dysf. vs. funct)
				0.013*
				1.000 (Dysf. vs. control)
				**0.010** (Funct. vs. control)
				0.008* (Dysf. vs. funct)

*BCs* Overall breast cancer survivors, *Dysf BCs* Metabolically dysfunctional BCs, *Funct BCs* Metabolically functional BCs, *SD* Standard deviation, *n* Number of participants, *kg* Kilogram, *mREE* Absolute resting energy expenditure, *mREE/FFM* FFM-adjusted resting energy expenditure, *mREE/FFMi* Fat free mass index-adjusted mREE, *FM* Fat mass (in kg and %), *FFM* Fat free mass (in kg and %), *FMi (FM/m*^*2*^*)* Fat mass index, *FFMi (FFM/m*^*2*^*)* Fat free mass index. *denotes significance at α < 0.05

FFM-adjusted mREE differed significantly between BCs and controls, with higher levels in the survivor group (BCs: 33 ± 3; vs. controls: 31 ± 3; *p* = 0.031). A trend towards significance was noticed when comparing Dysf BCs, Funct BCs and healthy controls (*p* = 0.081). FFM-adjusted mREE did not differ between Dysf and Funct (*p* = 0.576) (Table 2).

BW was not significantly different between BCs and healthy controls (BCs: 70.9 ± 14.9 vs. controls: 70.4 ± 13.8; *p* = 0.889). A significant difference in BW was present between Dysf BCs, Funct BCs and healthy controls (*p* = 0.008). A significant difference in BW was also seen between Dysf and Funct (Dysf>Funct; *p* = 0.002) (Table 2).

No significant differences were found for FM (kg,%) and FFM (kg,%) between BCs and controls. The difference in FMi and FFMi was also not significant. Significant differences in body composition became apparent between Dysf BCs, Funct BCs and healthy controls (FM_kg_: Dysf>controls>Funct; *p* = 0.040; FFM_kg_: Dysf>controls>Funct; *p* = 0.029). A significant difference in FM (kg,%) and FFM (kg) was also present between Dysf and Funct (FM_kg_: *p* = 0.002; FM_%_: *p* = 0.044; FFM_kg_: *p* = 0.013). Also for FMi and FFMi, significant differences are present with higher values in Dysf BCs (FMi: Dysf>controls>Funct; *p* = 0.041; FFMi: Dysf>controls>Funct); *p* = 0.013) (Table 2).

### Correlation analysis in BCs and healthy controls

In all BCs, a very strong positive correlation was seen between BW and FM (r = 0.943; *p* < 0.001), and between BW and FFM (r = 0.807; *p* < 0.001). A strong, positive correlation was found between FM and FFM (r = 0.582; *p* < 0.001). For the healthy controls, BW and FFM were strong and positively correlated(r = 0.722; *p* < 0.001), as well as BW and FM (r = 0.720; *p* < 0.001). A moderate, positive correlation was found between FM and FFM (r = 0.492; *p* = 0.002) (Table 3).

**Table 3:** Correlation analysis and linear regression analysis between metabolic parameters and body composition parameters for breast cancer survivors and healthy controls

BCs (*n* = 32)	FM	FFM	BW	mREE	Controls (*n* = 36)	FM	FFM	BW	mREE
**FM**					***FM***				
Pearson	1	0.582	0.943	0.513	*Pearson*	1	0.492	0.720	0.399
*p*-value		< 0.001*	< 0.001*	**0.003**	*p-value*		0.002*	< 0.001*	0.016*
**FFM**					***FFM***				
Pearson	0.582	1	0.807	0.715	*Pearson*	0.492	1	0.722	0.624
*p*-value	< 0.001*		< 0.001*	< 0.001*	*p-value*	0.002*		< 0.001*	< 0.001*
**BW**					***BW***				
Pearson	0.943	0.807	1	0.643	*Pearson*	0.720	0.722	1	0.713
*p*-value	< 0.001*	< 0.001*		< 0.001*	*p-value*	< 0.001*	< 0.001*		< 0.001*
**mREE**					***mRE*E**				
Pearson	0.513	0.715	0.643	1	*Pearson*	0.399	0.624	0.713	1
*p*-value	**0.003**	< 0.001*	< 0.001*		*p-value*	0.016*	< 0.001*	< 0.001*	
**Linear regression analysis for absolute mREE in BCs and controls**									
**BCs (n = 32)**	***R***^***2***^	***R***^***2***^ ***change***		***p-value***	***Controls (n = 36)***	***R***^***2***^	***R***^***2***^ ***change***		***p-value***
**mREE**					***mREE***				
Model:				< 0.001*	* Model:*				< 0.001*
FFM		0.511		< 0.001*	*BW*		0.508		< 0.001*
BW		0.013		0.385	*FFM*		0.025		0.192
FM	0.511	0.002		0.725	*FM*	0.508	0.024		0.194

*BCs* breast cancer survivors: *FM* fat mass: *FFM* fat free mass: *BW* body weight: *mREE* absolute resting energy expenditure: *mREE/FFM* FFM-adjusted resting energy expenditure adjusted. *denotes significance at α < 0.05

For all BCs, the strongest positive correlation for absolute mREE was found with FFM (r = 0.715; *p* < 0.001), followed by BW (*r* = 0.643; *p* < 0.001) and FM (*r* = 0.513; *p* = 0.003). In the control group, absolute mREE was positively correlated with BW (very strong: *r* = 0.713; *p* < 0.001) and FFM (strong; r = 0.624; *p* < 0.001). A moderate positive correlation was found with FM (r = 0.399; *p* = 0.016) (Table 3).

### Linear regression analysis of absolute mREE with body composition in BCs and healthy controls

A linear regression was calculated to predict absolute mREE based on FFM, FM and BW. For BCs, a significant regression equation was found for absolute mREE (F(3, 20)=10.333; *p* < 0.001), with *R*^2^ = 0.511. For the healthy controls, a significant regression equation was found for mREE (F(3, 32)=13.432; *p* < 0.001), with R^2^ = 0.508 (Table 3).

### Analysis of predictive variables for absolute mREE in BCs

After controlling for FFM and FM, a significant regression equation was found with survival year (F(6, 25)=8.192; *p* < 0.001) with R^2^ = 0.663 (no significant main effect). A significant interaction between survival year and FM was determined (F(2, 25)=3.825; *p* = 0.036 with η_p_^2^ = 0.234). The regression equation was also significant with being metabolically dysfunctional (F(3, 28)=10.495; p < 0.001) with *R*^2^ = 0.529, although no main effect was determined (Table 4).

**Table 4:** Predictive variables for absolute mREE in BCs

BCs (*n* = 32)	R^2^	η_p_^2^	*p*-value
**mREE**	0.663		< 0.001*
Survival year		0.134	0.148
FFM		0.308	0.003*
FM		0.035	0.347
Survival year*FM		0.234	0.036*
**mREE**	0.529		**< 0.001**
Metabolically dysfunctional		0.010	0.608
FFM		0.341	< 0.001*
FM		0.015	0.514

*BCs* Breast cancer survivors, *mREE* Absolute resting energy expenditure, *mREE/FFM *FFM-adjusted resting energy expenditure adjusted, *FFM* fat free mass, *FM* fat mass: η_p_^2^ = partial eta^2^. *denotes significance at α < 0.05

## Discussion

The present study was designed to examine the differences between absolute (mREE) and FFM-adjusted (mREE/FFM) measured energy expenditure and body composition (BW, FFM and FM) in breast cancer survivors (BCs) ≤5y post initial treatment compared to healthy, age-, BH- and BW matched controls without prior cancer diagnosis. The main findings are that BCs have similar absolute mREE, BW, FFM and FM compared to the control group, whereas FFM-adjusted mREE is significantly greater. Our data hereby suggest that BCs ≤5y post initial treatment have similar (absolute mREE) or greater (FFM-adjusted mREE) energy expenditure when compared to healthy controls without prior cancer diagnosis. The results of our study confirm the discrepancy between absolute and adjusted mREE, as previously observed by Madzima et al.(2020) in BCs >5y post treatment [20].

Absolute mREE is influenced by the body’s metabolically active components: FM and FFM [21]. Since a larger body size encompasses more FM and FFM, absolute mREE elevates accordingly [22]. The similarities in absolute weights of BW and body composition found in our study can explain the equal levels of mREE. Secondly, it can also be that breast cancer and -treatment may not lead to significant metabolic burden as compared to other tumors of more metabolically demanding organs as postulated by Nguyen and colleagues (2016) [23].

However, intra-individual differences in the distribution and proportion of organ tissue and -mass, each contributing individually to REE, makes it indispensable to normalize absolute mREE to FFM [24]. When adjusted to FFM, our study found greater mREE in BCs. Additionally, Vanittalie et al. suggested that a better analysis of the individual components can be made with FM and FFM normalized for BH (FMi and FFMi), as it allows comparison between individuals with different heights, taking the proportion of organ tissue into account [25]. When adjusting to FFMi, mREE is significantly greater in BCs compared to healthy controls, irrespective of being metabolically (dys)functional. Our findings suggests that BCs may experience more metabolic stress compared to a healthy control group, potentially due to the long-term metabolic effects of cancer or its therapy.

Significant deterioration of the metabolic profile in BCs is a known long-term complication of treatment [26, 27]. Concordantly, BCs endure treatment associated bodily changes including weight gain that most likely results from a net increase in FM, as pro-wasting mechanisms related to treatment (especially CT) facilitate loss of FFM [27–29]. According to Dieli-Conwright et al. (2022), an 8% increase in BW can be expected after treatment for breast cancer, with approximately a 17% increase in FM [2]. Further deterioration of FM can occur as BCs experience menopausal symptoms naturally persisting throughout survivorship, or prolonged by cancer-directed endocrine therapies [30]. Higher FM is related to the development of metabolic dysfunctions which seem to elevate mREE in a stepwise manner according to the number of dysfunctions [20, 31–36]. Our results show significantly higher BW and FM in Dysf BCs compared to the Funct BCs. In addition, absolute mREE elevates accordingly. After adjusting to FFM (and FFMi), however, no difference in energy expenditure was present. The precise energetic cost of the individual metabolic dysfunctions leading to altered mREE remains unknown, which can explain for the variability in absolute mREE between Dysf and Funct BCs [35]. From our study, we cannot determine the relationship between FM and metabolic dysfunctions, and an increase in absolute or adjusted mREE. Longitudinal research (progressing into survivorship) on larger sample sizes is necessary to examine the relationship between cancer treatment, deterioration of body composition and the onset of metabolic dysfunctions, and their impact on mREE.

### Predictive variables for absolute mREE in BCs

FFM was the single significant predictor of absolute mREE in BCs (explaining 51.1% of its variance), while BW was found to be the sole determinant in the control group (50.8%). For both BCs and controls, FFM significantly predicted FFM-adjusted mREE. We could not determine a main effect of survival year on absolute and adjusted mREE. However interestingly, a significant interaction between survival year and FM was seen for absolute mREE. Hyper−/hypometabolism had a main effect on absolute mREE, explaining ±30% of the variance. Being metabolically dysfunctional did not contribute to absolute mREE. The best prediction of absolute mREE was made with survival year, or hyper−/hypometabolism(both 66.3%).

The significant effect of the interaction between survival year and FM on absolute mREE can partially be explained by the fact that adult women naturally gain weight over time, as a mean weight increase of 1.06 kg – 2.38 kg per 4 year has been found [37]. More, BCs often undergo weight gain in terms of FM as a result of physical fatigue and often co-existing sedentarism experienced in the months or even years after diagnosis and treatment [38]. This makes BCs vulnerable for developing metabolic dysfunctions related FM [39]. Although we hypothesized a significant effect of being metabolically dysfunctional, no contribution to absolute mREE was determined, in contrast to previous research [2]. However, an hyper- or hypometabolism did add significantly to the prediction of absolute mREE when accounted for FFM and FM. Altered metabolism results in higher (hypermetabolism) or lower (hypometabolism) energy expenditure due to metabolic- and hemodynamic aberrations, and related changes in body composition [35]. In the absence of a deranged body composition profile, it can by suggested that hyper−/hypometabolism is caused (sub)clinical metabolic alterations [16].

### Limitations

We acknowledge several limitations. Despite our efforts to include a larger sample, we encountered difficulties in willingness of participants (time constraints, distance from home to research lab, psychological reasons). The results of our study should therefore be seen in light of research with similar objectives. Secondly, due to low sample sizes, we were unable to further investigate hormonal fluctuations, menopausal status, or estrogen suppression status. In addition, we did not control for meal consumption and physical activity. Third, since we do not have baseline and follow-up measures of body composition during and after treatment, we can only hypothesize the effects of CT, and concordant metabolic derangements. Finally, we retrospectively searched for the presence of metabolic dysfunctions in the aftermath of CT. Follow-up on metabolic derangements during and after treatment might provide more insights on fluctuations and interindividual variations in mREE. More research on a larger cohort is needed on (the onset of) metabolic dysfunctions and body composition in BCs after treatment and altered energy requirements to understand the mechanisms behind alterations in mREE.

## Conclusion

Our study reveals similar (absolute) or higher (FFM-adjusted) mREE in BCs ≤5y post active treatment compared to healthy controls. In absence of differences in body composition, we suggest that elevated mREE is the result of metabolic stress as a consequence of previous cancer diagnosis and/or treatment. Future research should continue to explore accurate FFM and FM (in relation to survival year) in relation to energy expenditure and metabolic health among BCs. Our study highlights the importance of accurate measurement of FFM and FM (in relation to survival year) in BCs. Precise measuring REE by IC is preferred when adapting nutritional strategies, especially in patients at risk for developing the metabolic syndrome since the individual contribution of the metabolic derangements to REE remains unknown and can lead to hyper−/hypometabolism.

### Supplementary Information

**Additional file 1: Table A. **Criteria for defining metabolic dysfunctions. **Table B. **Estrogen suppression therapy.

## Data Availability

The data supporting the findings of this study are available upon reasonable request from the corresponding author. Data are not shared openly to protect privacy of study participants. Data are located in a controlled access data storage at the University of Antwerp.
