# Supplementary material for: Resting energy expenditure, body composition, and metabolic alterations in breast cancer survivors vs. healthy controls: a cross-sectional study
Source: BMC Womens Health. 2024 Feb 12;24:117. doi: 10.1186/s12905-024-02900-y (PMC10863378; doi:10.1186/s12905-024-02900-y)
Supplement: Supplementary file 1 — Additional file 1: Table A. Criteria for defining metabolic dysfunctions. Table B. Estrogen suppression therapy. [file 12905_2024_2900_MOESM1_ESM.docx]

# **Supplementary tables**

**Table A.** Criteria for defining metabolic dysfunctions

| **Metabolic dysfunction**  Hypercholesterolemie  Hypertriglyceridemia  Hypertension  Insulin resistance  Increased adiposity  Increased blood glucose | **Criteria**  > 190 mg/dL  > 170 mg/dL  Systolic blood pressure ≥140 mmHg and/or diastolic blood pressure ≥90 mmHg  Blood glucose to insulin ratio < 4,5  FM>30% of total body weight with BMI>25.0 kg/m^2^  Fasted blood glucose > 125 mg/dL |
| --- | --- |

**Table B.** Estrogen suppression therapy

| **Estrogen suppression drug.** | |
| --- | --- |
| Tamoxifen  Anastrozol  Letrozol | N = 13  N = 7  N = 5  **Total = 25 patients** |
